# Is extended pelvic lymph node dissection REALLY required for staging of prostate cancer in the PSMA-PET era?

**DOI:** 10.1038/s41391-024-00821-3

**Published:** 2024-03-22

**Authors:** Matthew J. Roberts, John W. Yaxley, Johan Stranne, Inge M. van Oort, Derya Tilki

**Affiliations:** 1 Royal Brisbane and Women’s Hospital, Department of Urology, Brisbane, QLD Australia; 2 The University of Queensland, UQ Centre for Clinical Research, Brisbane, QLD Australia; 3 The University of Queensland, Faculty of Medicine, Brisbane, QLD Australia; 4 The Wesley Hospital, Brisbane, QLD Australia; 5 Department of Urology, Institute of Clinical Science, The Sahlgrenska Academy, University of Gothenburg, Gothenburg, Sweden; 6 Department of Urology, Region Västra Götaland, Sahlgrenska University Hospital, Gothenburg, Sweden; 7 Department of Urology, Radboud University Medical Center, Nijmegen, The Netherlands; 8 Martini-Klinik Prostate Cancer Center, University Hospital Hamburg Eppendorf, Hamburg, Germany; 9 Department of Urology, University Hospital Hamburg-Eppendorf, Hamburg, Germany; 10 Department of Urology, Koc University Hospital, Istanbul, Turkey

**Keywords:** Prostate cancer, Cancer therapy

An extended pelvic lymph node dissection (ePLND) at time of radical prostatectomy remains a recommended approach by most international prostate cancer guidelines. Enthusiasts for ePLND argue that it provides optimal staging compared to any imaging with possible oncological benefit and helps patient selection for early salvage therapies. The overall benefit of ePLND to a patient, when balancing the risks of complications and the absence of level 1 evidence of improved long term prostate cancer specific survival outcomes is uncertain. However, does knowledge of some, but not all, pelvic lymph node histopathology (N-stage) following an ePLND REALLY justify the additional risks?

Prospective trials have examined the performance of Prostate Specific Membrane Antigen (PSMA)-PET for lymph node staging compared to ePLND [1, 2]. PSMA-PET has a high specificity (95%) and positive predictive value (PPV; 87%) to detect lymph node metastases in high (specificity 93%, PPV 81%) and intermediate (specificity 96%, PPV 93%) risk patients [1, 2]. Positive lymph nodes on PSMA-PET can indicate greater histopathological lymph node burden than negative PSMA-PET and the incorporation of positive PSMA-PET findings into established nomograms has also been shown to improve their discriminative ability [3]. Therefore, a positive PSMA-PET adds value for patient management and may identify metastases outside the boundaries of an ePLND.

The impact of a false negative PSMA-PET result is a concern for clinicians. The sensitivity of PSMA-PET (40–58%) [1, 2] is higher than CT and MRI. Reassuringly, the negative predictive value (NPV) is 75–79%. Within both studies, the NPV (96%, 87%) was higher in intermediate risk patients. Novel nomograms may further enhance patient selection for ePLND. They have been shown to be applicable to all patients (regardless of MRI use) and demonstrate superior discriminative ability than older nomograms [4]. Furthermore, no metastases were found in patients with intermediate risk CAPRA scores, negative PSMA-PET and mpMRI PIRADS score of <5 with no mpMRI evidence of seminal vesicle invasion [5]. Therefore, combining negative PSMA-PET with clinical information [5] and nomograms (to indicate lymph node invasion prevalence) may serve to reduce unnecessary ePLND further [3, 4]. PSMA-PET lymph node staging performance in ***high-risk*** patients is sub-optimal (sensitivity 51%, NPV 73%) [1]. However, NPV is influenced by prevalence of lymph node invasion, where NPV of 99%, 87 and 84% are reported for prevalences of 5%, 20 and 40%, respectively. Therefore, the use of nomogram cutoffs to guide ePLND selection may be superseded by this multifaceted, bespoke approach for individual patients.

Gold standard staging should sample all metastasis sites, yet it has been clinically acceptable for the usual ePLND template to miss up to one third of pelvic metastases according to SPECT/CT/MRI with intraoperative gamma probe localisation [6]. Additionally, histopathological evaluation has limitations, detecting less positive lymph nodes than a combined molecular analysis (PSA qPCR within lymph nodes; 23% vs 52%) [7]. Within both studies, missed metastases were outside the usual ePLND template (common iliac 16–37%, para-aortic/caval 12%, pre-sacral/para-rectal 8%). PSMA-PET has confirmed anatomical limitations of ePLND, where up to 47% of patients with lymph node metastases would fall outside the ePLND template [8].

Technical expertise required for ePLND can also influence outcomes [9]. A sub-group comparison between pre-operative and post-operative PSMA-PET available for 37% of patients showed that 81% of patients with PSMA-PET suggestive of pelvic lymph node metastases on pre-operative staging had positive lymph nodes on restaging, of which 57% were persistent and 24% were recurrent to new pelvic sites [9]. A similar post-operative outcome for biochemical persistence after surgery was observed in the Hope study [2].

Use of ePLND as staging method carries morbidity, including perioperative complications (in up to 15% of patients), longer operating time, thromboembolic disease (6–10 fold increase), lymphocele formation, and potentially longer time in hospital [10]. Longer-term morbidity from the LAPPRO trial reported that patient reported moderate to severe lower limb and genital lymphoedema for 13.7% at 3 months (adjusted Risk Ratio 6.9) if they received ePLND causing significantly worse quality of life (adjusted for incontinence and erectile dysfunction). Symptoms remained at 12 and 24 months and were higher than doctor reported measures (4–5% for ePLND, 0.5–1.5% for no ePLND).

ePLND should not be mandated for any patients undergoing surgery, many factors should be considered in the PSMA PET era (Table 1).

**Table 1:** SWOT (strengths, weaknesses, opportunities, and threats) analysis of extended pelvic lymph node dissection (ePLND) in the Prostate-specific membrane antigen (PSMA) PET era.

Strengths	Weaknesses
• Confirm metastases not detected by imaging (false-negatives) • Accurate histopathological staging (false-positives) • Total tumour burden reduction via excision (if nodal metastases present)	• Limitation of anatomical template (missed metastases outside template) • Limitation of histopathology (may not detect all metastatic cells) • Associated morbidity/complications, impact on health and quality of life • Increased intra-operative time
**Opportunities**	**Threats**
• Incorporation with PSMA-PET imaging for targeted (Radioguided/Sentinel) LND • Improved patient selection considering prevalence of metastatic disease (higher nomogram cut-off)	• Absent Level 1 evidence for significant oncological benefit • Increasing demand on health services to limit low-value care/interventions • Surgeon proficiency/training • Earlier use of androgen receptor signalling inhibitors and other medications (pN status less relevant)

So, what is the optimal staging modality for prostate cancer patients? It is clear that risk calculators and PSMA-PET are complementary and should be used together to inform patient selection for ePLND. Therefore, we propose some alternative care pathways instead of routine ePLND.

A positive PSMA-PET for staging at diagnosis, may prompt a plan for post-operative pelvic lymph node radiotherapy, either after an ePLND to confirm pN1 or no ePLND if pN1 is assumed from the PSMA-PET. In this scenario, radical prostatectomy is largely for local control, until trials assessing the role of surgery in oligometastatic disease are available. Radiotherapy incorporating pelvic lymph nodes is likely to be more oncologically effective than ePLND (due to wider coverage outside surgical template), whilst limiting morbidity such as lymphoedema, especially in the absence of a prior ePLND [10].

For patients with a negative pre-operative PSMA-PET, an ePLND should not be mandatory, even in high risk disease. Instead, underlying lymph node invasion prevalence should be considered to guide decision-making. If predicted LNI prevalence is high, a negative PSMA-PET should prompt the discussion about the risks and benefits of ePLND due to sub-optimal detection of lymph node metastasis with both ePLND and PSMA-PET. Ideally, a post-operative plan made a priori, with adjuvant or early salvage radiotherapy to be considered in the event of post-operative PSA persistence or recurrence. For intermediate risk patients, (low prevalence) with a negative PSMA-PET, ePLND can be omitted with almost 90% confidence that significant metastases will not be missed [1], potentially with even higher confidence if a PIRADS < 5 lesion is present [5].

In the absence of contemporary level 1 evidence that early knowledge of the histological pelvic lymph node status improves outcomes with adjuvant therapy compared to an early-salvage approach, especially in a PSMA-PET triaged cohort, prospective trials are urgently needed to test these evolving paradigms due to incorporation of modern imaging already in clinical use.
